# Combined Synbiotics and Omega-3 Polyunsaturated Fatty Acids Enhance Clinical and Histological Recovery in DSS-Induced Ulcerative Colitis: An Experimental Study in Rats

**DOI:** 10.3390/diseases14060192

**Published:** 2026-05-29

**Authors:** Ioannis Varnalidis, Orestis Ioannidis, Athina Papadopoulou, Theofilos Poutahidis, Ioannis Taitzoglou, Aliki Brenta, Elissavet Anestiadou, Savvas Symeonidis, Stefanos Bitsianis, Ioannis Mantzoros, Manousos George Pramateftakis, Efstathios Kotidis, Stamatis Angelopoulos

**Affiliations:** 1 Medical School, Aristotle University of Thessaloniki, 54124 Thessaloniki, Greece; varnalidis@me.com (I.V.); athypa81@gmail.com (A.P.); 2 4th Department of General Surgery, General Hospital of Thessaloniki “G. Papanikolaou”, Aristotle University of Thessaloniki, 54124 Thessaloniki, Greece; alikibrenta@gmail.com (A.B.); elissavetxatz@gmail.com (E.A.); simeonidissavvas@yahoo.com (S.S.); sbitsiani@gmail.com (S.B.); imanvol@gmail.com (I.M.); mpramateftakis@hotmail.com (M.G.P.); skotidis@gmail.com (E.K.); angelopoulos.stamatis@gmail.com (S.A.); 3 Laboratory of Pathology, Faculty of Veterinary Medicine, Aristotle University of Thessaloniki, 54124 Thessaloniki, Greece; teoput@vet.auth.gr; 4 Laboratory of Physiology, Faculty of Veterinary Medicine, Aristotle University of Thessaloniki, 54124 Thessaloniki, Greece; jotai@vet.auth.gr

**Keywords:** ulcerative colitis, DSS colitis, synbiotics, probiotics, prebiotics, omega-3 fatty acids, rat model

## Abstract

Background/Objectives: Ulcerative colitis (UC) is a chronic inflammatory bowel disease in which alterations in the gut microbiota and dietary lipid composition play a central role; this study aimed to evaluate the effects of synbiotics, omega-3 polyunsaturated fatty acids, and their combination on clinical, macroscopic, microbiological, and histopathological outcomes in dextran sodium sulfate (DSS)-induced colitis in Wistar rats. Methods: Seventy-two male Wistar rats were randomly allocated to four groups (*n* = 18/group) and received 5% DSS in drinking water for eight days to induce colitis. Following DSS withdrawal and histological confirmation of colitis in sentinel animals, groups were treated for 8 days as follows: DSS (control), DSS-S (synbiotics, Ecologic^®^ 825), DSS-Ω3 (omega-3 fatty acid-enriched diet, ProSure^®^), or DSS-S&Ω3 (combined therapy). Eight rats per group were sacrificed on days 4 and 8 post-DSS. Body weight, Disease Activity Index (DAI), distal colon length, hematologic parameters, bacterial translocation to the liver and mesenteric lymph nodes, histological colitis score, and myeloperoxidase (MPO)-positive cell counts were assessed. Results: DSS induced severe colitis characterized by diarrhea, rectal bleeding, and extensive mucosal erosions. After 8 days of treatment, the DSS-S&Ω3 group showed the greatest body-weight recovery (206.1→222.9 g, *p* < 0.05 vs. other groups), significantly preserved distal colon length, and the largest reduction in DAI (*p* < 0.05). Both the DSS-S and DSS-S&Ω3 groups demonstrated reduced bacterial translocation compared with DSS. The DSS-Ω3 group demonstrated persistent MPO-positive neutrophil infiltration compared with the DSS-S and DSS-S&Ω3 groups, whereas combined therapy was associated with lower MPO-positive cell counts. Histological colitis scores were significantly improved only in the DSS-S&Ω3 group (*p* < 0.05). Conclusions: In this DSS colitis model, the DSS-S&Ω3 group demonstrated superior clinical and histological outcomes compared with DSS-S or DSS-Ω3 alone, supporting further evaluation of combined synbiotic and omega-3 therapy as an adjunctive approach in ulcerative colitis.

## 1. Introduction

Ulcerative colitis (UC) is a chronic idiopathic inflammatory bowel disease characterized by relapsing inflammation of the colonic mucosa [1,2]. The etiology involves interactions among genetic predisposition, host immunity, gut microbiota, and environmental factors [3,4,5]. Environmental influences, particularly dietary patterns, are increasingly recognized as significant contributors to intestinal dysbiosis and disruption of the mucosal barrier in IBD. Recent work demonstrates that high consumption of ultra-processed foods is associated with altered gut microbial composition and attenuated inflammatory responses that exacerbate disease activity in ulcerative colitis [6]. Disruptions in microbial homeostasis (dysbiosis) contribute to immune activation and epithelial barrier dysfunction, central features of UC pathogenesis [7,8,9,10,11].

Conventional therapeutic strategies—5-aminosalicylic acid compounds, corticosteroids, immunomodulators, and biological agents—achieve remission in many patients but remain limited by inadequate response rates and adverse effects [12,13,14,15]. Consequently, interest has grown in adjunctive therapies that modulate gut microbiota, reduce inflammation, and support mucosal healing.

Synbiotics, combining probiotics and prebiotics, can promote the growth of beneficial bacterial populations, improve epithelial barrier integrity, reduce inflammatory cytokine production, and decrease bacterial translocation [16,17,18,19,20]. Clinical studies demonstrate that synbiotics may induce remission and reduce inflammatory markers in UC [21,22,23]. Preclinical studies similarly show reduced histological inflammation and decreased MPO activity following synbiotic administration [24,25].

Omega-3 polyunsaturated fatty acids modulate inflammatory responses by altering eicosanoid profiles, decreasing production of pro-inflammatory cytokines, and promoting resolution-phase mediators [26,27,28,29,30,31]. Clinical trials have shown mixed but generally favorable effects on disease activity and steroid-sparing potential in UC [28,29,30,31,32,33,34,35]. Experimental studies also reveal attenuation of colitis severity and reductions in inflammatory mediators with ω3 fatty acid supplementation [36,37,38].

Previous studies have shown that omega-3 fatty acids and synbiotics, each compared with tap water, exert distinct but complementary beneficial effects in dextran sodium sulfate (DSS)-induced experimental ulcerative colitis [39,40]. However, their combined therapeutic potential has not been adequately investigated. Given their potentially complementary mechanisms—microbiota modulation and anti-inflammatory action—it is plausible that co-administration may result in additive or synergistic effects. Therefore, this study aimed to determine whether combined synbiotic and omega-3 fatty acid therapy provides superior clinical, macroscopic, microbiological, and histopathological outcomes compared with either intervention alone in DSS-induced colitis in Wistar rats.

## 2. Materials and Methods

### 2.1. Animals and Ethical Approval

Seventy-two male Wistar rats aged approximately 2.5 months were obtained from an accredited breeding facility. Animals were obtained and maintained under specific pathogen-free (SPF) conditions and were housed in groups under controlled environmental conditions (temperature 20–22 °C, relative humidity 55–65%, 12 h light/dark cycle) during both the DSS administration period and the subsequent treatment phase, with free access to standard chow and tap water. Following acclimatization, rats weighed approximately 210–230 g. All procedures complied with European Directive 86/609/EEC for the protection of animals used for scientific purposes and strictly followed the ARRIVE (Animal Research: Reporting of In Vivo Experiments) guidelines to ensure high standards of study design, reporting, and reproducibility [38].

### 2.2. Randomization and Blinding

Following a seven-day acclimatization period, each rat was weighed and assigned a unique identification code. Randomization was performed using a computer-generated sequence in R with a fixed seed to ensure reproducibility. To minimize baseline variability, animals were stratified by body weight prior to allocation. Rats were ranked by weight and divided into four equal blocks, after which animals were allocated according to the randomization sequence from each block into one of four groups (n = 18 per group). Following DSS administration, two randomly selected animals from each group (total n = 8) were euthanized for histological confirmation of successful colitis induction. The remaining 16 animals per group subsequently entered the treatment phase and were equally distributed into two subgroups (n = 8 per group) for evaluation at day 4 and day 8 post-DSS.

The primary outcome for sample size estimation was the histological colitis score at day 8 post-treatment, as this endpoint directly reflects mucosal injury and recovery. The Disease Activity Index (DAI) was considered an important secondary clinical outcome reflecting overall disease severity. An a priori sample-size calculation was performed for the primary endpoint using G*Power software (version 3.1, Heinrich Heine University Düsseldorf, Germany) for one-way ANOVA comparing four independent groups. Assuming a moderate-to-large effect size (f = 0.40), a significance level of α = 0.05, and statistical power of 80%, the required sample size was estimated at approximately 8 animals per group per time point. The effect-size assumption was based on previously published DSS-induced colitis studies reporting comparable differences in histological inflammation and clinical disease activity following synbiotic or omega-3 interventions [14,18,19,39,40]. Accordingly, 8 animals per group per time point was considered sufficient to detect biologically meaningful differences in histological recovery, while DAI was analyzed as an important secondary clinical outcome.

Group allocation was performed by a researcher not involved in treatment administration or outcome assessment. To minimize cage effects, animals from each treatment group were distributed across multiple cages rather than housed by treatment. Investigators responsible for histological evaluation and immunohistochemical analysis were blinded to group allocation throughout the study.

### 2.3. Induction of Experimental Colitis

Experimental colitis was induced by administering 5% dextran sodium sulfate (DSS; molecular weight 36,000–50,000 Da, MP Biomedicals, Santa Ana, CA, USA), which was dissolved in drinking water at a concentration of 5% and administered ad libitum for 8 days. Animals were monitored daily for general clinical condition and fluid consumption. Although exact individual water and food intake measurements were not recorded, all cages were inspected daily to ensure adequate access to water and diet throughout the experimental period.

DSS induces colonic epithelial injury, crypt distortion, mucosal ulceration, and neutrophil infiltration and closely mimics the histopathological features of acute ulcerative colitis [41,42,43]. During DSS administration, animals were monitored daily for stool consistency, presence of diarrhea, and visible rectal bleeding. After eight days, DSS was withdrawn and replaced with tap water for the remainder of the experiment.

To confirm successful induction of colitis and document baseline mucosal injury, two randomly selected rats from each group were euthanized immediately after DSS cessation. Distal colon samples were collected and examined histologically to confirm epithelial erosions, crypt abnormalities, and inflammatory infiltrates characteristic of DSS-induced colitis.

### 2.4. Experimental Groups and Treatment Interventions

Following DSS withdrawal, the remaining rats (n = 16 per group) were assigned to one of the following treatment regimens for eight days:

DSS (control): Animals received standard laboratory chow and tap water without any additional synbiotic or omega-3 supplementation.

DSS-S (synbiotics): Rats received a synbiotic formulation (Ecologic^®^ 825, Winclove Bio Industries, Amsterdam, The Netherlands) administered once daily by oral gavage. The formulation contained multiple probiotic strains, including *Bifidobacterium bifidum*, *B. infantis*, *B. lactis*, *B. longum*, *Lactobacillus acidophilus*, *L. casei*, *L. plantarum*, *L. salivarius*, and *Lactococcus lactis*, combined with the prebiotics inulin and fructo-oligosaccharides. The probiotic dose administered was 2 × 10^8^ CFU per rat per day, diluted in sterile water and administered once daily.

DSS-Ω3 (omega-3 fatty acids): Rats received an omega-3 fatty acid–enriched nutritional formulation (ProSure^®^, Abbott Laboratories, Abbott Park, IL, USA) administered once daily by oral gavage. The formulation contained eicosapentaenoic acid (EPA) and docosahexaenoic acid (DHA) at concentrations previously reported to modulate inflammatory responses in experimental colitis models. Omega-3 supplementation was administered at a dose comparable to that previously used in DSS-induced experimental colitis studies (approximately 200 mg/kg/day total omega-3 fatty acids), daily by oral gavage in sterile water.

DSS-S&Ω3 (combination therapy): Rats received both the synbiotic formulation and the omega-3-enriched diet once daily. The administration of the two components was separated by approximately one hour to optimize absorption and minimize potential formulation interactions.

Eight animals from each group were euthanized on day 4 post-DSS to assess early post-treatment effects, and the remaining eight animals were euthanized on day 8 to evaluate late recovery-phase changes and mucosal healing.

**Figure 1:**
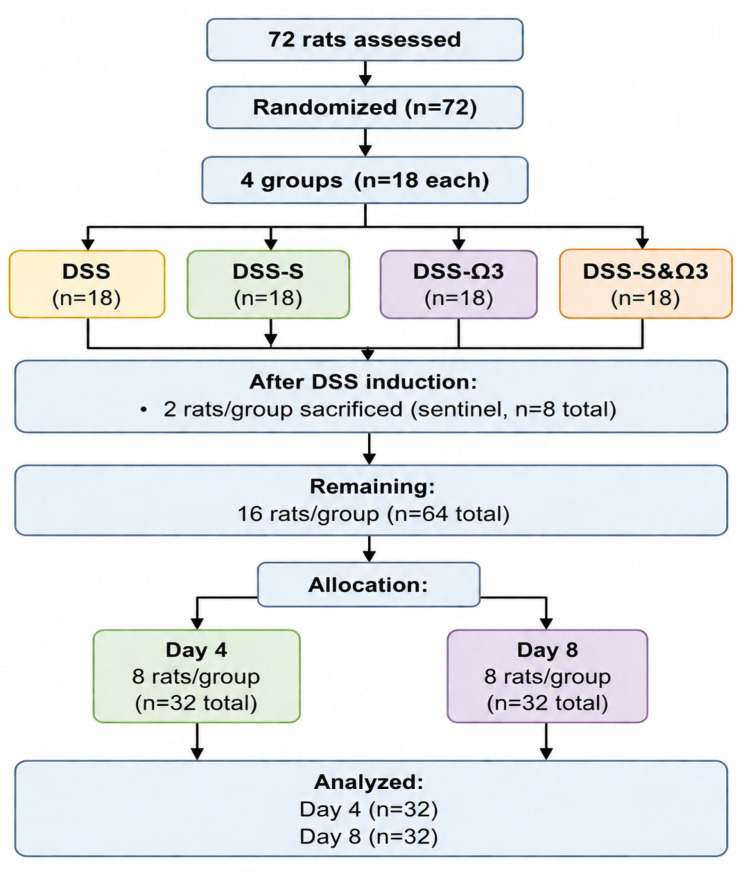
Schematic flow diagram of animal allocation, follow-up, and analysis in the DSS-induced experimental colitis model. Initially, 72 rats were randomized into four groups (n = 18/group). Following DSS induction, two animals per group were sacrificed for histological confirmation of colitis (sentinel animals; n = 8 total). The remaining animals entered the treatment phase and were equally allocated for evaluation at day 4 and day 8 post-treatment (n = 8/group per time point).

A schematic flow diagram of animal allocation, follow-up, and analysis is presented in Figure 1.

### 2.5. Clinical Assessment

Body weight was recorded daily throughout DSS exposure and the treatment period. Stool characteristics (normal, loose, diarrhea) and rectal bleeding (absent, occult, visible) were assessed daily. These parameters, together with percentage body-weight loss, were incorporated into the Disease Activity Index (DAI), a validated scoring system widely used in DSS colitis models to quantify clinical severity [22]. Body-weight loss was scored as follows: 0 = no weight loss, 1 = 1–5%, 2 = 5–10%, 3 = 10–20%, and 4 = >20% weight loss. Relative body-weight recovery (%) was calculated relative to body weight at treatment initiation following DSS withdrawal. Stool consistency was scored as 0 = normal stool, 2 = loose stool, and 4 = diarrhea, while rectal bleeding was scored as 0 = no bleeding, 2 = occult bleeding, and 4 = gross bleeding. The final DAI score was calculated as the mean of the three component scores, according to previously established DSS colitis scoring systems [44].

### 2.6. Euthanasia and Tissue Collection

Animals were deeply anesthetized with intraperitoneal chloral hydrate. Euthanasia was performed by cardiac puncture followed by exsanguination. A midline laparotomy was performed to expose abdominal organs. The entire colon was resected, gently flushed with saline to remove fecal contents, and measured from the ileocecal junction to the rectum. The distal colon was weighed, photographed, and examined for macroscopic abnormalities, including erythema, edema, strictures, erosions, and ulcerations. Specifically, a standardized distal colonic segment approximately 2–3 cm proximal to the rectum was collected from all animals for histopathological and immunohistochemical analysis.

Segments of the distal colon were fixed in 10% neutral buffered formalin for histopathological and immunohistochemical analysis. The distal colon was specifically selected because DSS-induced inflammatory and histopathological alterations are typically most severe and consistently reproducible in this anatomical region, making it the most commonly evaluated site in experimental DSS colitis models. Additional samples from the liver and mesenteric lymph nodes were collected aseptically for microbiological evaluation of bacterial translocation.

### 2.7. Hematologic Evaluation

Blood samples obtained by cardiac puncture were collected into EDTA tubes and analyzed immediately using an automated hematology analyzer. Parameters assessed included red blood cell count, hemoglobin concentration, hematocrit, mean corpuscular volume (MCV), mean corpuscular hemoglobin (MCH), and platelet count. Peripheral blood smears were stained with Giemsa for morphological evaluation.

### 2.8. Microbiological Assessment of Bacterial Translocation

Liver and mesenteric lymph node samples were homogenized under sterile conditions in phosphate-buffered saline. Serial dilutions were plated onto selective and non-selective media, including Columbia blood agar, MacConkey agar, chocolate agar, Chapman agar, and CDC anaerobic agar. Plates were incubated under aerobic or anaerobic conditions as appropriate. After 24–48 h, colony-forming units (CFUs) per gram of tissue were counted, and representative colonies were identified using VITEK^®^ 2 and API biochemical testing systems. Representative cultured microorganisms and colony-forming unit (CFU) counts isolated from liver and mesenteric lymph node (MLN) samples on day 8 following treatment initiation in the DSS, DSS-S, DSS-Ω3, and DSS-S&Ω3 groups. The listed microorganisms represent detectable cultured isolates under the selected laboratory conditions and do not constitute a complete characterization of all microorganisms present within the tissues.

### 2.9. Histopathology

Formalin-fixed colon samples were embedded in paraffin, sectioned at 4–6 μm, and stained with hematoxylin and eosin. Histological scoring was performed using a validated grading system assessing:

- Epithelial damage (surface erosion, ulceration);
- Crypt architecture (dilatation, shortening, loss);
- Goblet cell depletion;
- Inflammatory cell infiltration (extent and depth);
- Mucosal thickness and edema.

Scores ranged from 0 (normal) to 4 (severe colitis), with scoring performed by an investigator blinded to the treatment group.

**Figure 2:**
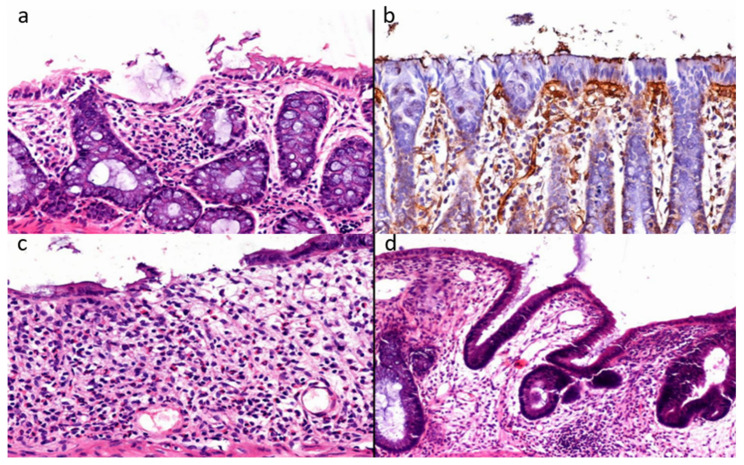
(**a**–**d**) Representative baseline histopathological findings confirming successful induction of DSS-induced experimental colitis. (**a**) Extensive erosion of the surface epithelium accompanied by dense inflammatory cell infiltration within the lamina propria. (**b**) Immunohistochemical staining of the epithelial basement membrane demonstrating focal epithelial microerosions with disruption of basement membrane continuity. (**c**) Advanced mucosal injury is characterized by epithelial erosion and severe crypt loss (glandular atrophy). (**d**) Mucinous atrophy with dysplastic and dilated colonic glands, goblet cell depletion, and epithelial pseudostratification. Panels (**a**,**c**,**d**) were stained with hematoxylin and eosin. Panel (**b**) represents immunohistochemistry using 3,3′-diaminobenzidine chromogen with hematoxylin counterstaining. Scale bars: (**a**–**c**) 50 μm; (**d**) 100 μm.

Histological colitis scoring was performed using a semi-quantitative scale based on epithelial damage, crypt architectural distortion, goblet cell depletion, inflammatory cell infiltration, mucosal thickness, and edema. Each parameter was graded from 0 to 4, where 0 indicated normal histological appearance, 1 indicated mild changes, 2 indicated moderate changes, 3 indicated marked changes, and 4 indicated severe colitis-related injury [45]. The final histological colitis score for each animal was calculated as the overall composite score based on these parameters, with higher scores indicating more severe mucosal injury. Scoring was performed by an investigator blinded to treatment allocation. Representative histopathological findings following DSS exposure are shown in Figure 2 and demonstrate the characteristic epithelial and inflammatory alterations confirming successful induction of experimental colitis.

### 2.10. Immunohistochemistry for MPO-Positive Cells

Additional sections were processed for immunohistochemical detection of myeloperoxidase (MPO), a marker of neutrophil infiltration. Sections underwent deparaffinization, antigen retrieval, and incubation with rabbit anti-MPO primary antibody. Staining was visualized using a polymer-HRP detection system with DAB chromogen, and slides were counterstained with hematoxylin.

Digital images were captured from ten randomly selected high-power fields per sample. MPO-positive cells were quantified manually or semi-automatically using ImageJ (version 1.54; National Institutes of Health, Bethesda, MD, USA; https://imagej.nih.gov/ij/) image analysis software.

### 2.11. Statistical Analysis

All statistical analyses were conducted using SPSS software (version 22.0). Continuous variables (body weight, colon length, hematologic indices) were expressed as mean ± standard deviation. Group comparisons were performed using one-way ANOVA followed by the least significant difference (LSD) post hoc test when ANOVA showed significance. Nonparametric variables (histological scores, MPO-positive cell counts, bacterial translocation data) were analyzed using the Kruskal–Wallis test, followed by pairwise Mann–Whitney U tests with Bonferroni correction.

A *p*-value < 0.05 was considered statistically significant. Statistical assumptions, including normality and homogeneity of variance, were tested prior to analysis.

Post hoc power analysis: A post hoc observed power analysis was conducted for selected continuous outcomes with available one-way ANOVA results, based on a design of four groups with eight animals per group at each time point (total N = 32, α = 0.05). The estimated statistical power was 0.92 for hemoglobin, 0.83 for distal colon length, and 0.80 for platelet count, indicating adequate power to detect moderate-to-large between-group differences for these endpoints. Post hoc power was not calculated for nonparametric outcomes (histological scores, MPO-positive cell counts, and bacterial translocation) due to the absence of distributional parameters required for such analyses.

## 3. Results

### 3.1. Clinical Observations and General Condition

Throughout the DSS administration period, all animals developed clinical signs consistent with acute colitis. Rats exhibited progressive weight loss, reduced activity, piloerection, and alterations in stool consistency. Diarrhea typically appeared by day 4 of DSS exposure, followed by visible rectal bleeding in most animals by days 6–8. The severity and timing of symptom onset were comparable across groups prior to treatment allocation, confirming uniform induction of colitis and validating the randomization process.

Following DSS withdrawal and initiation of treatment, clinical recovery varied depending on the intervention. Animals in the DSS-S&Ω3 group demonstrated the earliest and most pronounced improvement in grooming behavior, mobility, and stool consistency, whereas animals in the DSS group showed slower and incomplete recovery within the 8-day treatment period.

### 3.2. Body-Weight Changes

No significant differences in body weight were observed between groups at baseline. Body-weight loss was observed in all animals during DSS administration. During the subsequent 8-day treatment period, weight recovery differed between groups. Rats in the DSS group showed only minimal improvement, with mean body weight increasing from 193.3 g to 197.8 g, indicating a persistent inflammatory burden. The DSS-S group demonstrated more effective recovery, while the DSS-Ω3 group showed a more modest increase.

**Figure 3:**
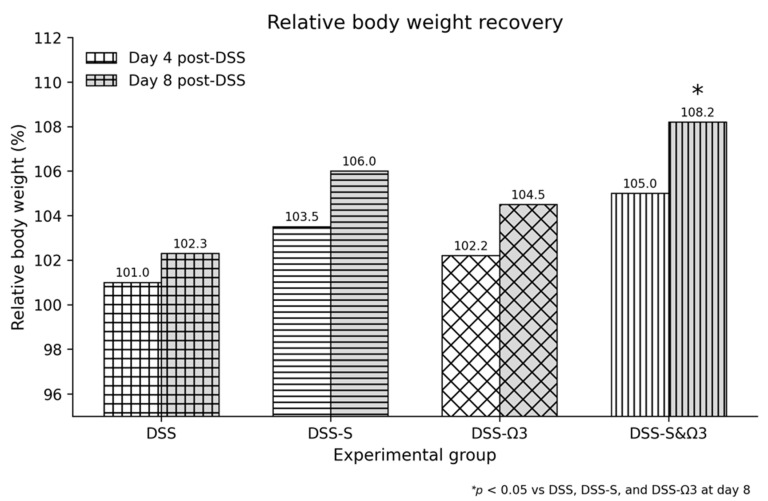
Relative body weight recovery (%) at days 4 and 8 following treatment initiation (p.t.), showing the greatest improvement in the DSS-S&Ω3 group compared with all other groups. Relative body-weight recovery (%) was calculated as: [(body weight at evaluation day − body weight at treatment initiation)/body weight at treatment initiation] × 100, where treatment initiation was defined as day 0 following DSS withdrawal and histological confirmation of colitis. Data are presented as mean values. * *p* < 0.05 versus DSS, DSS-S, and DSS-Ω3 at day 8.

**Figure 4:**
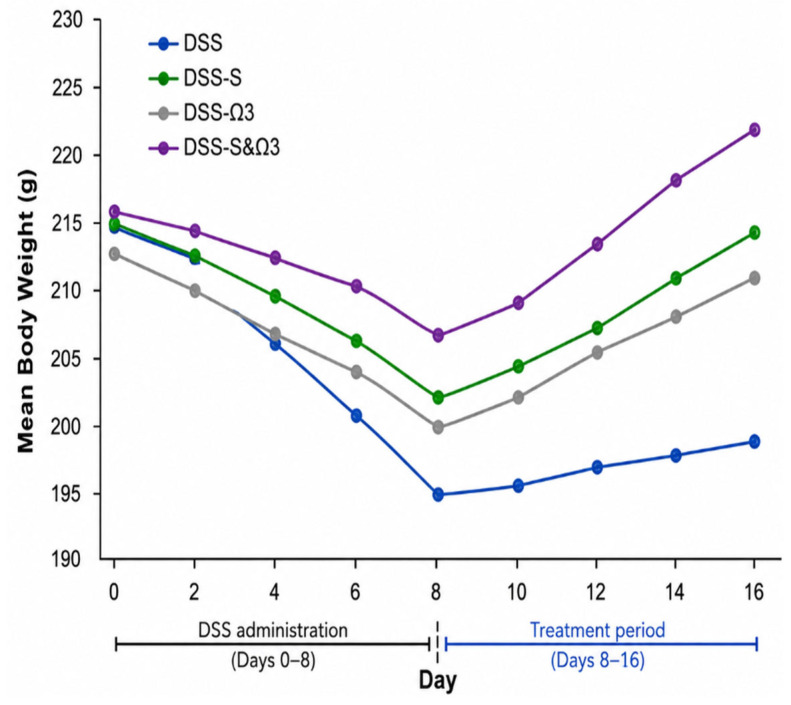
Mean body weight of rats prior to and following treatment initiation (blue: DSS; green: DSS-S; gray: DSS-Ω3; purple: DSS-S&Ω3). Mean body weight of rats before and after treatment initiation. Values are presented as estimated marginal means derived from the ANOVA model. Body weight values at baseline (prior to treatment initiation) did not differ significantly between groups (*p* > 0.05), indicating successful randomization despite minor numerical variation. Data are shown for descriptive and comparative purposes.

The most substantial and consistent improvement was observed in the DSS-S&Ω3 group, where body weight increased significantly from 206.1 g to 222.9 g, reflecting accelerated clinical recovery. This increase was statistically significant compared with the DSS, DSS-S, and DSS-Ω3 groups at day 8 (*p* < 0.05) (Figure 3 and Figure 4). Detailed numerical values and statistical comparisons for relative body-weight recovery are presented in Table 1.

### 3.3. Disease Activity Index (DAI)

Disease Activity Index (DAI) scores increased progressively during the eight-day DSS exposure period in all animals, reflecting the development of clinically significant colitis. By the final day of DSS administration, most rats exhibited severe diarrhea, overt rectal bleeding, and substantial weight loss, resulting in peak DAI scores that were comparable across all groups at baseline.

Following treatment initiation, DAI decreased in all groups; however, the rate and magnitude of improvement varied. In the DSS group, the reduction in DAI was slow and incomplete, with many animals continuing to exhibit loose stools and intermittent bleeding at day 8. The DSS-S group demonstrated a more pronounced decrease in DAI, with earlier resolution of diarrhea and reduction in rectal bleeding compared with DSS. The DSS-Ω3 group showed a moderate decline in DAI, although several animals maintained higher bleeding scores during the early recovery phase, suggesting persistent mucosal vulnerability.

**Figure 5:**
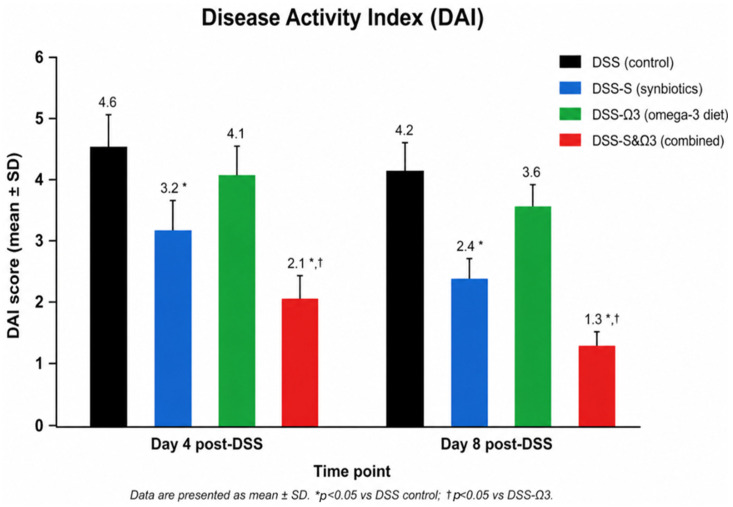
Disease Activity Index (DAI) scores on days 4 and 8 following treatment initiation in the DSS, DSS-S, DSS-Ω3, and DSS-S&Ω3 groups. Day 0 was defined as the time of DSS withdrawal and histological confirmation of colitis. Data are presented as mean ± SD. * *p* < 0.05 versus DSS control; † *p* < 0.05 versus DSS-Ω3.

The most rapid and substantial improvement was observed in the DSS-S&Ω3 group, where animals demonstrated early clinical recovery, with several rats achieving near-normal stool consistency by day 6 and minimal residual bleeding. Both the DSS-Ω3 and DSS-S&Ω3 groups showed a statistically significant reduction in DAI compared with baseline (*p* < 0.05), with the greatest and most consistent improvement observed in the DSS-S&Ω3 group (Figure 5). This rapid clinical improvement parallels the observed acceleration in body-weight recovery and histological repair. Detailed numerical values and statistical comparisons for the primary outcomes are summarized in Table 2.

### 3.4. Hematologic Parameters

**Figure 6:**
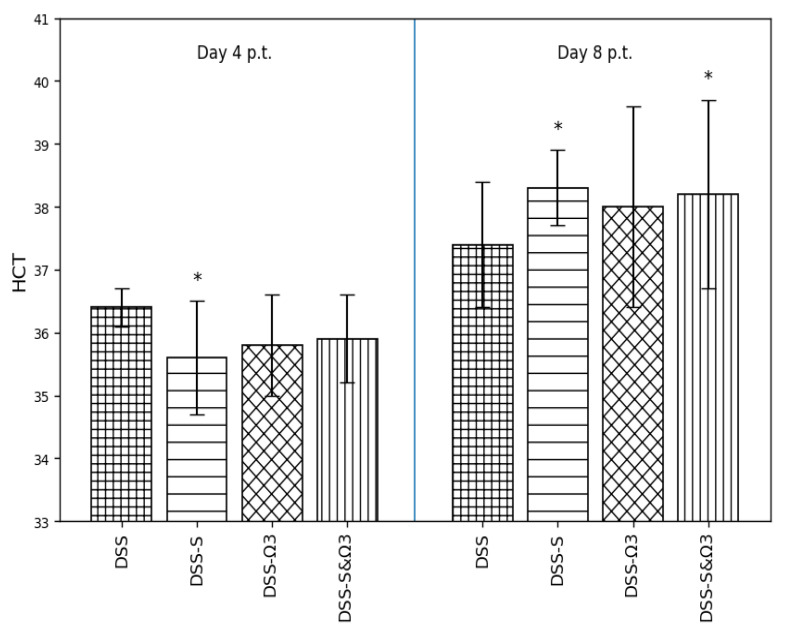
Comparison of hematocrit (HCT) values among the DSS, DSS-S, DSS-Ω3, and DSS-S&Ω3 groups on days 4 and 8 following treatment initiation. Data are presented as mean ± standard deviation. Statistically significant differences compared with DSS are indicated by * (*p* < 0.05).

**Figure 7:**
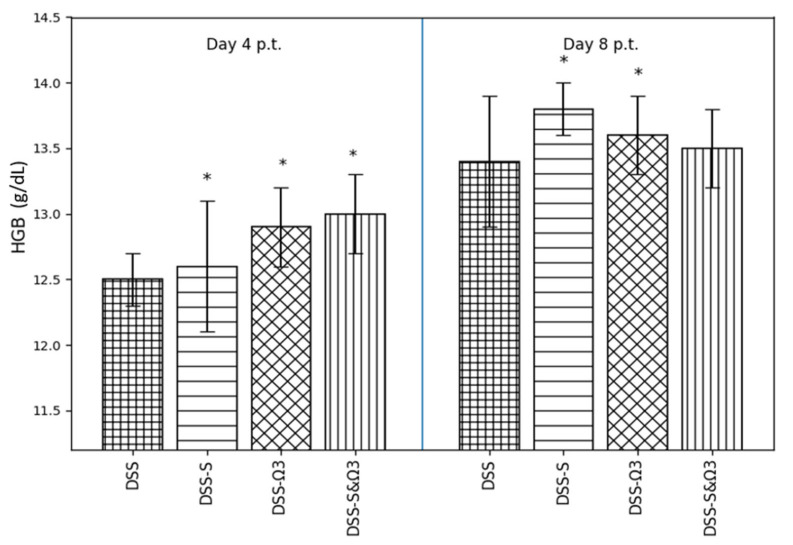
Mean ± standard error of hemoglobin (HGB) levels on days 4 and 8 following treatment initiation in the DSS, DSS-S, DSS-Ω3, and DSS-S&Ω3 groups. Significant differences between groups are indicated by * (*p* < 0.05).

**Figure 8:**
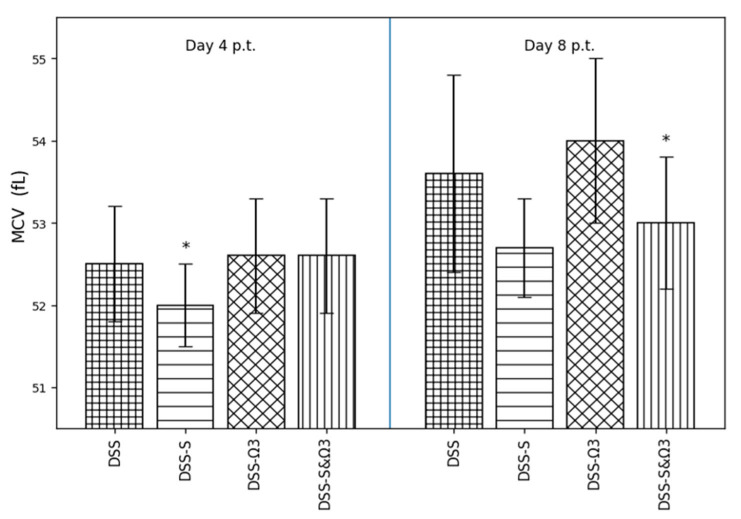
Mean ± standard error of mean corpuscular volume (MCV) on days 4 and 8 following treatment initiation in the DSS, DSS-S, DSS-Ω3, and DSS-S&Ω3 groups. * *p* < 0.05 versus DSS.

**Figure 9:**
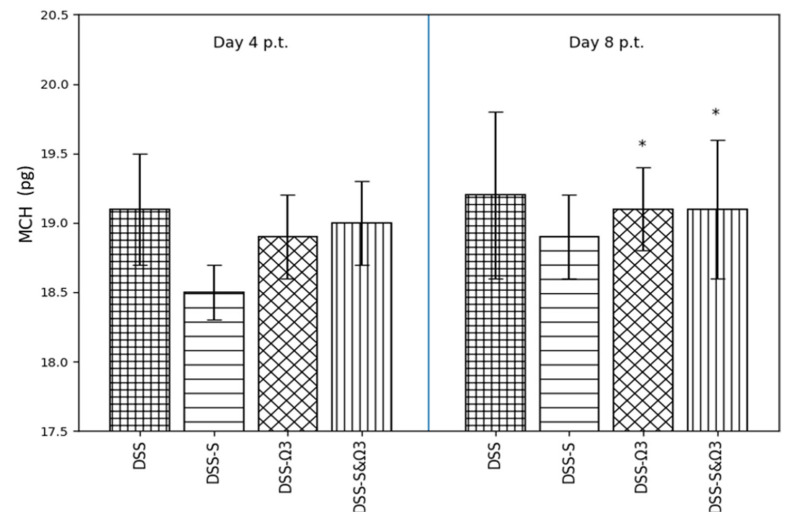
Mean ± standard error of mean corpuscular hemoglobin (MCH) on days 4 and 8 following treatment initiation in the DSS, DSS-S, DSS-Ω3, and DSS-S&Ω3 groups. * *p* < 0.05 versus DSS.

**Figure 10:**
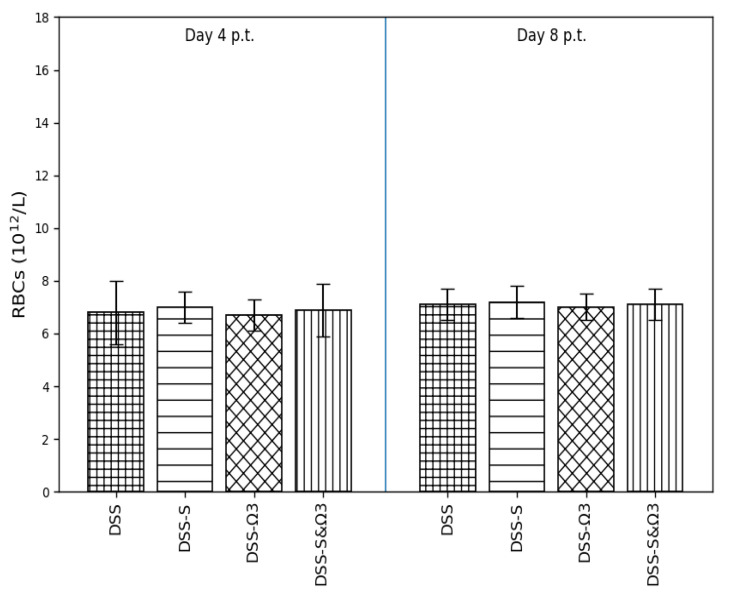
Mean ± standard error of red blood cell (RBC) count on days 4 and 8 following treatment initiation in the DSS, DSS-S, DSS-Ω3, and DSS-S&Ω3 groups. No statistically significant differences were observed between groups.

**Figure 11:**
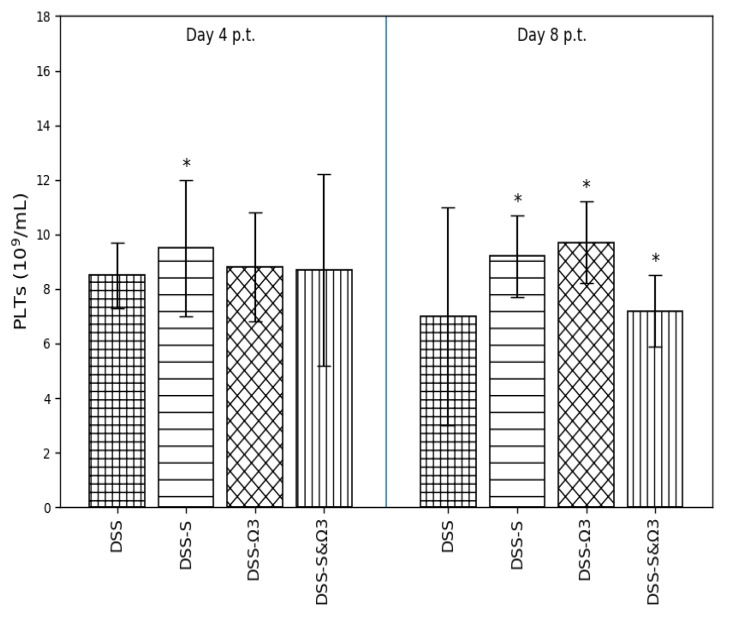
Mean ± standard error of platelet (PLTs) count on days 4 and 8 following treatment initiation in the DSS, DSS-S, DSS-Ω3, and DSS-S&Ω3 groups. Statistically significant differences compared with DSS are indicated by * (*p* < 0.05).

Hematologic analysis revealed anemia and thrombocytosis in all groups following DSS exposure. During treatment, hemoglobin concentrations increased significantly in the DSS-S, DSS-Ω3, and DSS-S&Ω3 groups compared with the DSS group (one-way ANOVA, *p* = 0.0062), suggesting reduced blood loss and improved systemic recovery (Figure 6, Figure 7, Figure 8, Figure 9, Figure 10 and Figure 11). Hematologic parameters were evaluated on days 4 and 8 following treatment initiation.

#### 3.4.1. Hematocrit (HCT)

Hematocrit values (Figure 6) showed a modest reduction in the DSS group at day 4. Treatment with synbiotics (DSS-S), ω-3 fatty acids (DSS-Ω3), and their combination (DSS-S&Ω3) partially attenuated this decrease. By day 8, HCT levels were significantly increased in the DSS-S group compared with DSS (*p* < 0.05), while the DSS-Ω3 and DSS-S&Ω3 groups also exhibited higher values, indicating progressive recovery over time.

#### 3.4.2. Hemoglobin (HGB)

Hemoglobin levels (Figure 7) followed a similar pattern. At day 4, HGB values were slightly higher in all treatment groups compared with DSS, with the greatest increase observed in the DSS-Ω3 and DSS-S&Ω3 groups. By day 8, HGB levels were significantly elevated in the DSS-S and DSS-Ω3 groups compared with DSS (*p* < 0.05), indicating improved erythropoietic status.

#### 3.4.3. Mean Corpuscular Volume (MCV)

Mean corpuscular volume (Figure 8) remained relatively stable at day 4 across all groups, with a modest reduction observed in the DSS-S group. At day 8, MCV values were significantly increased in the DSS-Ω3 group compared with DSS (*p* < 0.05), while the DSS-S&Ω3 group showed a similar upward trend, suggesting a potential effect of ω-3 fatty acids on erythrocyte volume during recovery.

#### 3.4.4. Mean Corpuscular Hemoglobin (MCH)

Mean corpuscular hemoglobin values (Figure 9) did not differ significantly between groups at day 4. However, at day 8, MCH was significantly higher in the DSS-Ω3 and DSS-S&Ω3 groups compared with DSS (*p* < 0.05), reflecting improved hemoglobin content per erythrocyte following ω-3 supplementation.

#### 3.4.5. Red Blood Cell Count (RBC)

Red blood cell counts (Figure 10) remained stable across all groups at both time points, with no statistically significant differences observed between DSS and treatment groups. These findings indicate that changes in hemoglobin-related parameters were not primarily driven by alterations in erythrocyte number.

#### 3.4.6. Platelet Count (PLTs)

Platelet counts (Figure 11) were increased in the DSS-S group at day 4 compared with DSS (*p* < 0.05). At day 8, PLT levels remained significantly higher in both the DSS-S and DSS-Ω3 groups relative to DSS (*p* < 0.05), whereas the DSS-S&Ω3 group demonstrated partial normalization. Notably, platelet counts in the combination group were significantly lower than in the DSS group at day 8 (*p* = 0.0241), consistent with attenuation of inflammation-associated thrombocytosis.

### 3.5. Bacterial Translocation to Liver and Mesenteric Lymph Nodes

Bacterial translocation was prominent at baseline, with high numbers of aerobic and anaerobic organisms cultured from liver and mesenteric lymph node samples across all groups. Table 3 presents representative microorganisms isolated following DSS exposure, prior to treatment initiation. Culture-based analysis focused on detectable and clinically relevant bacterial isolates; therefore, the listed organisms do not necessarily represent the complete microbial composition. Detection limits were determined according to standard microbiological culture sensitivity thresholds.

The most pronounced improvement was observed in the DSS-S&Ω3 group, where several animals exhibited no detectable aerobic bacterial growth by day 8, and overall microbial recovery was characterized by low-virulence anaerobes at markedly reduced densities (Table 4).

Overall, bacterial translocation was significantly reduced in the DSS-S and DSS-S&Ω3 groups compared with DSS (*p* < 0.05), with the lowest colony-forming unit (CFU) counts observed in the DSS-S&Ω3 group. These findings suggest that synbiotics, particularly when combined with omega-3 fatty acids, more effectively enhance epithelial barrier integrity than omega-3 treatment alone.

### 3.6. Distal Colon Length

DSS exposure resulted in substantial shortening of the distal colon in all groups, consistent with acute inflammatory injury and muscular contracture. At the time of DSS withdrawal, the degree of shortening was comparable across groups, indicating uniform baseline disease severity.

By day 8 of treatment, distal colon length differed significantly between groups (one-way ANOVA, *p* = 0.0191). The DSS group exhibited minimal recovery, maintaining a markedly reduced colon length. The DSS-S group showed greater elongation of the distal colon, suggesting reduced mucosal edema and partial resolution of inflammation. The DSS-Ω3 group demonstrated variable recovery, with partial elongation in some animals but persistent shortening in others.

**Figure 12:**
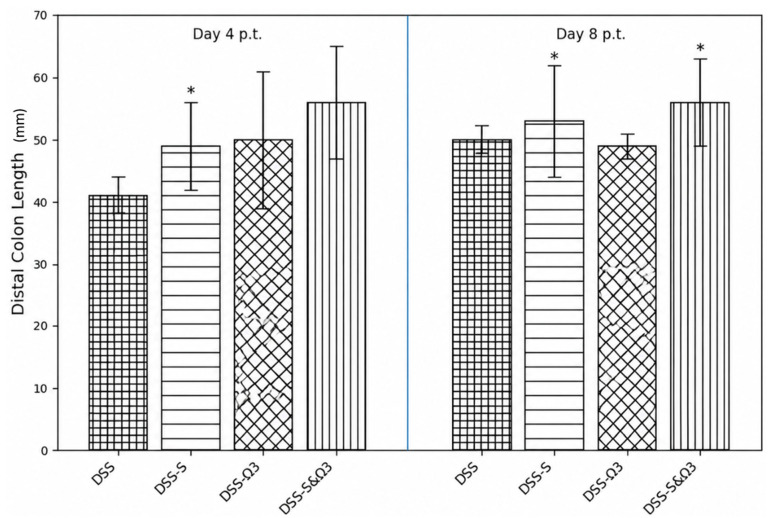
Distal colon length at day 4 and day 8 following treatment initiation in the DSS, DSS-S, DSS-Ω3, and DSS-S&Ω3 groups. Distal colon length was measured in millimeters (mm) following sacrifice. Data are presented as mean ± standard error. Measurements were evaluated relative to baseline (day 0) values obtained at treatment initiation. * *p* < 0.05 versus DSS. Preservation of colon length was greater in the DSS-S&Ω3 group compared with DSS and DSS-Ω3 groups, consistent with reduced colonic inflammation and tissue damage.

The most pronounced recovery was observed in the DSS-S&Ω3 group, which demonstrated the greatest preservation and restoration of distal colon length compared with all other groups (Figure 12). These findings indicate that combined synbiotic and omega-3 therapy was most effective in reversing DSS-induced colon shortening.

### 3.7. Macroscopic Evaluation of the Colon

Macroscopic examination following DSS withdrawal revealed severe colonic inflammation in all groups, characterized by hyperemia, edema, and friable mucosa with widespread erosions. These findings were consistent across groups at baseline, confirming uniform induction of severe colitis.

By day 4 of treatment, colons from the DSS group remained markedly inflamed, with diffuse erythema, wall thickening, and persistent erosions. Animals in the DSS-S group exhibited reduced hyperemia and fewer erosive lesions compared with DSS, while the DSS-Ω3 group showed mild improvement in mucosal appearance but continued to display prominent erosions.

By day 8, the DSS group continued to demonstrate extensive erosions and mucosal thickening, with minimal structural recovery. The DSS-S group showed further macroscopic improvement, with a reduction in both the number and severity of erosions. The DSS-Ω3 group demonstrated partial recovery but remained inferior to DSS-S, with several animals still exhibiting large erosive areas.

The most pronounced macroscopic recovery was observed in the DSS-S&Ω3 group. By day 8, these colons exhibited markedly reduced hyperemia and near-complete resolution of erosive lesions. The mucosal surface appeared smoother and more continuous, and wall thickness was substantially reduced compared with all other groups. These findings paralleled the observed improvement in body-weight recovery and were consistent with the significant improvements in clinical indices and histological outcomes.

### 3.8. Histopathological and Immunohistochemical Findings

Histological scoring was performed by a blinded investigator. Histopathological assessment confirmed severe epithelial injury, crypt disruption, goblet cell depletion, and dense inflammatory infiltrates at baseline in all groups, indicating uniform induction of colitis.

By day 4, animals in the DSS-S and DSS-S&Ω3 groups exhibited early signs of tissue repair, including partial re-epithelialization and reduced inflammatory cell infiltration, whereas the DSS group continued to demonstrate extensive mucosal damage.

**Figure 13:**
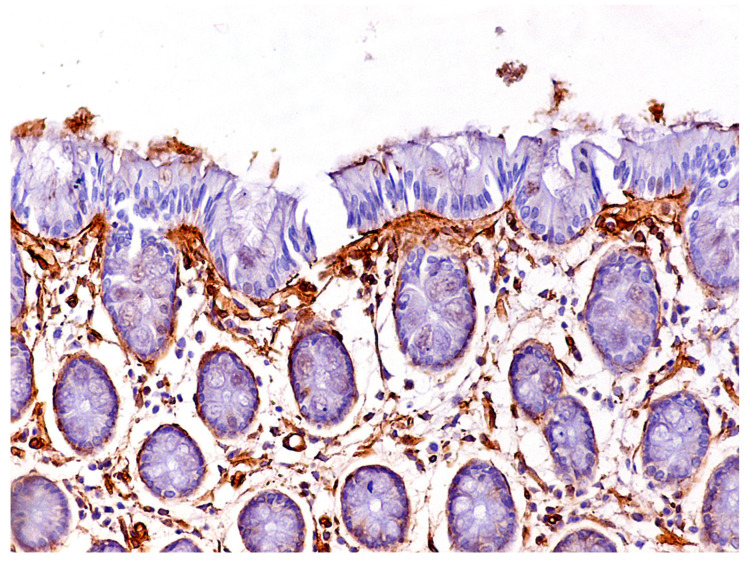
DSS-S&Ω3, day 8 following treatment initiation. Immunohistochemical staining for laminin (Laminin Ab-1; Thermo Fisher Scientific/Labvision, Fremont, CA, USA), demonstrating basement membrane integrity.

**Figure 14:**
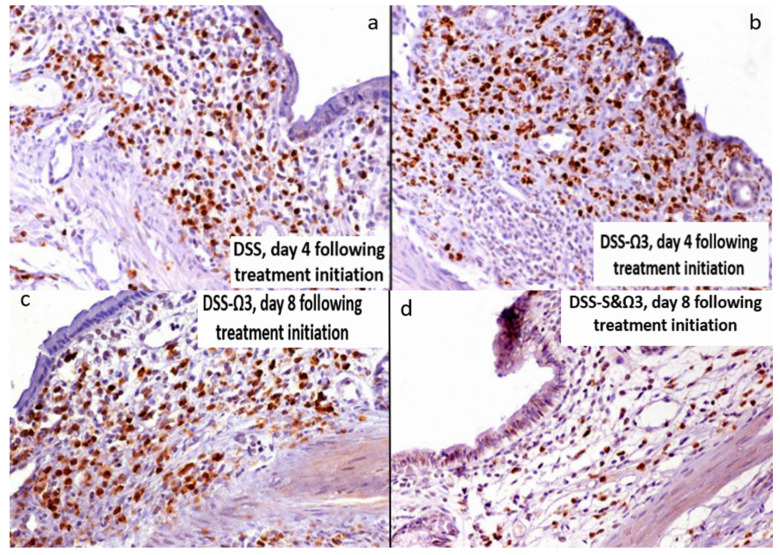
Myeloperoxidase (MPO) immunohistochemical staining of distal colon sections at days 4 and 8 post-treatment. (**a**) DSS group, day 4 post-treatment, showing extensive neutrophil infiltration within the mucosa. (**b**) DSS-Ω3 group, day 4 post-treatment, demonstrating persistent MPO-positive cell infiltration. (**c**) DSS-Ω3 group, day 8 post-treatment, showing sustained and increased MPO-positive cell infiltration compared with other groups. (**d**) DSS-S&Ω3 group, day 8 post-treatment, demonstrating markedly reduced MPO-positive cells and improved mucosal architecture. Overall, MPO staining highlights pronounced neutrophil infiltration following DSS exposure, which persists and is increased in the DSS-Ω3 group at day 8, whereas combined synbiotic and omega-3 treatment (DSS-S&Ω3) results in substantial attenuation of inflammatory cell infiltration.

**Figure 15:**
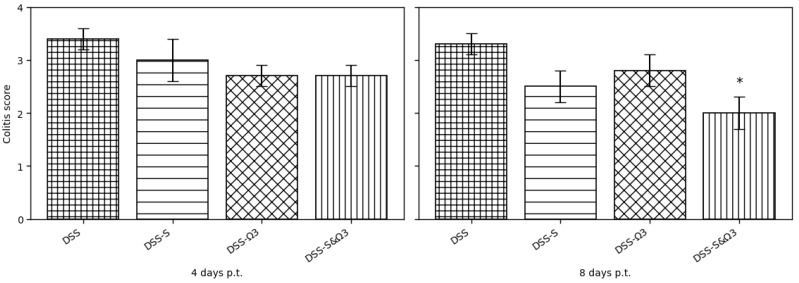
Histological colitis scores on days 4 and 8 following treatment initiation in the DSS, DSS-S, DSS-Ω3, and DSS-S&Ω3 groups. The most pronounced improvement in mucosal architecture was observed in the DSS-S&Ω3 group. Data are presented as mean ± SD. * *p* < 0.05 versus DSS, DSS-S, and DSS-Ω3 at the corresponding time point.

By day 8, the DSS group still showed pronounced epithelial erosions and crypt distortion. The DSS-S group demonstrated moderate histological improvement, while the DSS-Ω3 group exhibited partial reconstruction of epithelial architecture despite persistent abnormalities. In contrast, the DSS-S&Ω3 group showed the most advanced histological recovery (Figure 13 and Figure 14), characterized by restored epithelial continuity, improved crypt morphology, and significantly reduced inflammatory infiltration within the lamina propria. This corresponded to the lowest histological colitis scores among all groups (Figure 15). Histological scores were significantly reduced only in the DSS-S&Ω3 group compared with all other groups (*p* < 0.05).

**Figure 16:**
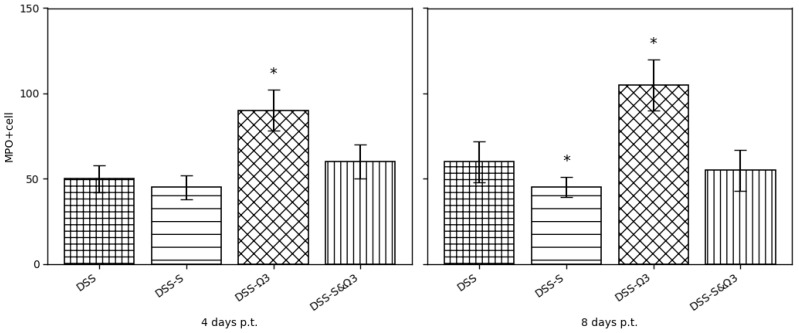
Quantification of MPO-positive neutrophil counts in distal colon sections at days 4 and 8 post-treatment. Data are presented as mean ± standard deviation. The DSS-Ω3 group exhibited significantly increased MPO-positive cell counts at day 8 compared with DSS, DSS-S, and DSS-S&Ω3 groups. In contrast, the DSS-S&Ω3 group demonstrated the lowest level of neutrophil infiltration, indicating enhanced resolution of inflammation. * *p* < 0.05 versus DSS, DSS-S, and DSS-S&Ω3 at the corresponding time point.

Immunohistochemical analysis of MPO-positive cells demonstrated extensive neutrophil infiltration in all groups at baseline. The DSS-Ω3 group exhibited significantly increased MPO-positive cell counts at day 8 compared with DSS, DSS-S, and DSS-S&Ω3 groups (*p* < 0.05). In contrast, the DSS-S group showed reduced MPO staining, while the DSS-S&Ω3 group demonstrated the most pronounced decrease in MPO-positive cells (Figure 16), consistent with enhanced resolution of neutrophil-driven inflammation. MPO-positive cell counts remained significantly lower in the DSS-S&Ω3 group compared with DSS and DSS-Ω3 (*p* < 0.05). 

## 4. Discussion

Previous studies have investigated the effects of individual nutritional interventions in DSS-induced experimental ulcerative colitis, including omega-3 polyunsaturated fatty acids and synbiotics, each compared with tap water as control [39,40]. Omega-3 fatty acid supplementation was previously associated with improvement in selected hematologic and clinical parameters, despite increased colonic neutrophil infiltration [39], whereas synbiotic administration was associated with attenuation of mucosal inflammation, reduced neutrophil infiltration, and improvement in selected laboratory and macroscopic outcomes [40]. These complementary findings provided the rationale for evaluating their combined administration in the present study.

Building on these previously published findings, the present study was designed to investigate whether combined synbiotic and omega-3 fatty acid therapy confers additive or synergistic therapeutic benefits in DSS-induced colitis. The results demonstrate that the DSS-S&Ω3 group showed superior recovery compared with DSS, DSS-S, and DSS-Ω3 across multiple outcome domains, including clinical activity, body-weight recovery, distal colon length, bacterial translocation, histological injury, and neutrophil infiltration. Taken together, these findings suggest that simultaneous modulation of gut microbiota and inflammatory pathways may produce a broader therapeutic effect than either intervention alone.

The present study demonstrates that the combination of synbiotics and omega-3 polyunsaturated fatty acids exerts a substantially greater therapeutic effect than either treatment alone in a DSS-induced model of ulcerative colitis. This synergistic benefit was reflected across multiple domains, including clinical recovery, weight restoration, improvement of stool characteristics, normalization of inflammatory hematologic markers, attenuation of bacterial translocation, and enhanced macroscopic and histological mucosal healing. Together, these findings provide compelling experimental evidence that dual modulation of the gut microbiota and inflammatory lipid pathways produces a more comprehensive anti-inflammatory response than targeting either mechanism independently.

Synbiotics have been increasingly recognized as promising modulators of intestinal homeostasis due to their ability to restore microbial balance, enhance short-chain fatty acid production, reinforce epithelial barrier integrity, and regulate immune responses [14,15,16,17,18,22,23,24,44,45,46,47]. Several clinical and experimental studies have reported improved outcomes in ulcerative colitis following synbiotic administration, including reduced mucosal inflammation, decreased pro-inflammatory cytokine expression, and amelioration of histological injury [5,19,20,21,22,23,24,48,49,50,51]. In the present study, the DSS-S group demonstrated moderate improvement in DAI, macroscopic appearance, and bacterial translocation, supporting previous observations that probiotics and prebiotics can strengthen barrier function and reduce luminal antigen exposure. However, synbiotics alone did not fully reverse the epithelial damage induced by DSS, suggesting that microbiota modulation, although beneficial, may require complementary anti-inflammatory mechanisms to achieve more complete mucosal recovery.

Omega-3 polyunsaturated fatty acids, particularly eicosapentaenoic acid (EPA) and docosahexaenoic acid (DHA), have been extensively studied for their ability to modulate inflammatory processes through alterations in membrane phospholipid composition, suppression of pro-inflammatory eicosanoids derived from arachidonic acid, and downregulation of cytokine production [25,26,27]. Clinical studies have reported variable but generally favorable effects of ω3 supplementation in ulcerative colitis, including reduced corticosteroid requirements and attenuation of relapse rates in selected patient populations [32,33,34,35]. Experimental studies have similarly demonstrated reductions in pro-inflammatory mediators such as TNF-α and leukotriene B4, as well as modulation of neutrophil activity following ω3 administration [10,36,37,38,47]. In the present study, the DSS-Ω3 group showed partial improvement in clinical parameters and epithelial repair. However, the DSS-Ω3 group also demonstrated relatively higher MPO-positive neutrophil counts at later time points compared with the DSS-S and DSS-S&Ω3 groups. Because baseline pre-treatment MPO quantification was not available, these findings should not be interpreted as definitive evidence of aggravated inflammation relative to the initial DSS-induced state. Rather, the persistence of MPO-positive cells may reflect delayed resolution or slower clearance of neutrophilic infiltration during the recovery phase. Previous studies have shown that ω3 fatty acids can modulate neutrophil recruitment and activation in a context-dependent manner, resulting in both anti-inflammatory and pro-resolving, but occasionally persistent, neutrophilic responses during active inflammation [10,11,27,47]. This residual neutrophilic activity may partially explain the less complete mucosal restoration observed in the DSS-Ω3 group, suggesting that although ω3 fatty acids effectively modulate inflammatory signaling pathways, they may not be sufficient alone to fully resolve epithelial injury without concurrent regulation of microbial and barrier-related mechanisms [29,30,31].

The principal finding of the present study is that the combination of synbiotics and omega-3 polyunsaturated fatty acids produced synergistic therapeutic effects compared with either intervention alone. Animals in the DSS-S&Ω3 group demonstrated the most rapid and consistent improvement in DAI, the greatest body-weight recovery, and the most pronounced macroscopic normalization of the colon. Histologically, this group exhibited extensive epithelial restoration, improved crypt architecture, and markedly reduced inflammatory infiltration. In addition, combination therapy resulted in the greatest reduction in bacterial translocation, supporting the concept that interventions targeting both microbial balance and epithelial barrier integrity are particularly effective when combined with agents that modulate inflammatory signaling. Notably, synbiotics may attenuate the persistent neutrophil activity observed in the DSS-Ω3 group, thereby contributing to a more coordinated resolution of inflammation.

The biological plausibility of this synergistic effect is supported by complementary mechanisms. Synbiotics promote the growth of beneficial microbial populations, enhance short-chain fatty acid production, and improve epithelial barrier function through increased mucin production and tight junction integrity [5,14,15,16,17,18,48]. Recent evidence further supports the role of synbiotics and microbiota-targeted interventions in modulating intestinal immune homeostasis, improving epithelial barrier integrity, and regulating inflammatory signaling pathways [10,47]. In parallel, ω3 fatty acids modulate inflammatory pathways and promote the generation of specialized pro-resolving mediators, facilitating resolution of inflammation [10,25,26,27,47]. These mechanisms target interconnected pathways involved in ulcerative colitis pathophysiology, including microbial dysbiosis, epithelial barrier disruption, and immune dysregulation. Simultaneous modulation of these pathways may, therefore, create a more favorable environment for mucosal healing, as reflected by the superior outcomes observed in the DSS-S&Ω3 group [52,53].

The DSS model used in this study reproduces key histopathological and immunological features of human ulcerative colitis, including mucosal ulceration, crypt damage, neutrophil infiltration, and increased intestinal permeability [38,39,40]. Although no experimental model fully captures the complexity of human disease, the consistency of the present findings across multiple outcome measures supports the robustness and reproducibility of the observed therapeutic effects. Importantly, the interventions evaluated in this study—synbiotics and omega-3 polyunsaturated fatty acids—are readily translatable into clinical practice, given their established safety profiles, accessibility, and prior use in patients with inflammatory bowel disease [41,42,43,46,49]. These characteristics support the potential clinical applicability of combined nutritional strategies as adjunctive approaches in ulcerative colitis management [18,19,20,21,22,41,42,43,51].

## 5. Strengths, Limitations and Future Perspectives

This study has several important strengths. A key advantage is its comprehensive and integrative experimental design, which enabled the simultaneous evaluation of clinical, macroscopic, hematologic, microbiological, histopathological, and immunohistochemical outcomes. This multidimensional approach provides a robust assessment of disease activity and treatment response, reducing the likelihood that the observed effects are attributable to isolated or method-specific findings. In addition, strict randomization, investigator blinding during histological and immunohistochemical analyses, and adherence to ARRIVE guidelines enhance the internal validity and reproducibility of the results. Notably, this study addresses an important gap in the literature by systematically evaluating the combined use of synbiotics and omega-3 polyunsaturated fatty acids in experimental ulcerative colitis, providing novel evidence of additive or synergistic therapeutic effects compared with monotherapy. Additionally, post hoc power analysis demonstrated adequate statistical power for selected continuous outcomes, including hemoglobin (0.92), distal colon length (0.83), and platelet count (0.80), supporting the robustness of the findings.

Despite these strengths, several limitations should be acknowledged. Only male Wistar rats were included in this study, which may limit the generalizability of the findings. Sex-based differences in immune responses, gut microbiota composition, and inflammatory pathways have been reported and may influence both disease severity and treatment response. Therefore, the present study does not account for potential sex-related variability. Future studies should include female cohorts to better elucidate sex-specific effects and improve the translational relevance of these findings. An additional limitation of the present study is the absence of a healthy, untreated control group. Consequently, baseline levels of bacterial translocation, mucosal integrity, and inflammatory cell infiltration in non-colitic animals could not be directly evaluated. However, the primary objective of the study was to compare the relative therapeutic effects of synbiotics, omega-3 fatty acids, and their combination within a standardized DSS-induced colitis model rather than to contrast diseased and healthy animals. Future studies incorporating healthy controls would allow more comprehensive characterization of baseline physiological and microbiological parameters. The DSS model represents an acute chemically induced form of colitis and does not fully replicate the chronic, relapsing nature of human ulcerative colitis, thereby limiting conclusions regarding long-term therapeutic effects. Furthermore, certain quantitative parameters—including detailed longitudinal DAI values, precise morphometric measurements of colon length, and standardized MPO quantification—were not available for advanced statistical modeling and effect size estimation. In addition, mechanistic analyses such as cytokine profiling, tight junction protein assessment, lipid mediator pathways, oxidative stress markers, and microbiome sequencing were not performed, limiting insight into the biological mechanisms underlying the observed effects. The relatively short duration of the post-treatment period (eight days) also restricts evaluation of sustained mucosal healing and relapse prevention. In addition, microbiological assessment relied on conventional culture-based and biochemical identification methods rather than molecular sequencing techniques, such as 16S rRNA analysis or metagenomic profiling, potentially limiting comprehensive characterization of gut microbial diversity, dysbiosis patterns, and bacterial translocation. Therefore, potential treatment-related alterations in fecal or colonic microbiota composition could not be fully assessed. Future studies incorporating molecular microbiome analyses would provide important mechanistic insight into the interaction between dysbiosis, intestinal inflammation, and therapeutic response.

From a translational perspective, these findings support further investigation of combination strategies that simultaneously target gut microbiota composition, epithelial barrier integrity, and inflammatory signaling pathways. Future studies should incorporate longer follow-up periods and chronic or relapsing models of colitis to better reflect the clinical course of ulcerative colitis. Inclusion of mechanistic analyses, including cytokine profiling and microbiome characterization, would further clarify the pathways underlying the observed synergistic effects. Given the favorable safety profile and widespread clinical availability of synbiotics and omega-3 fatty acids, these results provide a strong rationale for translational research and controlled clinical trials evaluating their combined use as an adjunct to standard therapy. If confirmed in human studies, this approach may represent a feasible and low-risk strategy to enhance mucosal healing and improve long-term disease management in ulcerative colitis.

## 6. Conclusions

Combined synbiotics and ω3 polyunsaturated fatty acids exert synergistic therapeutic effects in DSS-induced colitis, outperforming monotherapy across clinical, microbiological, and histopathological domains. These findings provide a strong rationale for clinical evaluation of combined nutritional strategies as adjunctive therapies in ulcerative colitis.

## Figures and Tables

**Table 1:** Relative body weight (%) at day 4 and day 8 following treatment initiation.

Group	Day 4 Post-DSS (% Mean ± SD)	Day 8 Post-DSS (% Mean ± SD)
DSS	101.0 ± 2.1	102.3 ± 2.5
DSS-S	103.5 ± 2.4	106.0 ± 2.8 *
DSS-Ω3	102.2 ± 2.3	104.5 ± 2.6
DSS-S&Ω3	105.0 ± 2.5 *	108.2 ± 2.9 *

* *p* < 0.05 versus DSS at the corresponding time point. Data are presented as mean ± standard deviation (SD).

**Table 2:** Summary of primary outcome measures (Disease Activity Index and histological colitis score) at days 4 and 8 following treatment initiation. Data are presented as mean ± standard deviation (SD).

Group	Day 4 DAI (Mean ± SD)	Day 4 Histological Score (Mean ± SD)	Day 8 DAI (Mean ± SD)	Day 8 Histological Score (Mean ± SD)
DSS	4.5 ± 0.5	6.0 ± 0.6	4.1 ± 0.5	5.8 ± 0.6
DSS-S	3.2 ± 0.4	4.5 ± 0.5	2.4 ± 0.3	4.0 ± 0.5
DSS-Ω3	4.1 ± 0.5	5.0 ± 0.6	3.6 ± 0.4	4.5 ± 0.5
DSS-S&Ω3	2.1 ± 0.3	3.5 ± 0.4	1.3 ± 0.2	2.0 ± 0.4
Statistical comparison	*p* < 0.05	*p* < 0.05	*p* = 0.012	*p* = 0.008

DAI: Disease Activity Index. Higher scores indicate more severe clinical or histological disease activity.

**Table 3:** Representative cultured microorganisms and colony-forming unit (CFU) counts isolated from liver and mesenteric lymph node (MLN) samples at the end of DSS exposure, prior to treatment initiation. The listed microorganisms represent detectable cultured isolates and do not constitute a complete characterization of the tissue microbiome.

Liver	Count (CFU/g Tissue)	MLN	Count (CFU/g Tissue)
*Bacillus* spp.	0.85 × 10^2^	*Pseudomonas putida*	2.5 × 10^2^
*Staphylococcus hominis*	1.04 × 10^2^	*Streptococcus viridans*	1.16 × 10^2^
*Candida* spp.	0.52 × 10^2^	*Staphylococcus coagulase-negative*	1 × 10^3^
*Staphylococcus epidermidis*	0.75 × 10^2^	*Streptococcus milleri*	1 × 10^3^
		*Staphylococcus warneri*	1.58 × 10^2^
		*Acinetobacter lwoffii*	1.58 × 10^2^
		*Escherichia coli*	1.08 × 10^2^

By day 4 of treatment, the DSS group maintained high bacterial loads with a diverse microbial profile. The DSS-S group demonstrated a marked reduction in bacterial translocation, with fewer pathogenic isolates recovered. The DSS-Ω3 group showed a moderate decrease in bacterial counts, although variability between animals was observed.

**Table 4:** Microbial population (colony-forming units per gram of tissue) of the liver and mesenteric lymph nodes (MLN) in the DSS, DSS-S, DSS-Ω3, and DSS-S&Ω3 groups on day 8 following treatment initiation. Representative cultured microorganisms and colony-forming unit (CFU) counts isolated from liver and mesenteric lymph node (MLN) samples on day 8 following treatment initiation in the DSS, DSS-S, DSS-Ω3, and DSS-S&Ω3 groups. The listed microorganisms represent detectable cultured isolates under the selected laboratory conditions and do not constitute a complete characterization of all microorganisms present within the tissues.

Group	Tissue	Microorganism	Count (CFU/g Tissue)
DSS	Liver	*Fusobacterium* spp.	1.2 × 10^3^
DSS	MLN	*Peptostreptococcus* spp.	0.86 × 10^2^
DSS-Ω3	Liver	*Peptostreptococcus tetradius*	8 × 10^2^
DSS-Ω3	Liver	*Fusobacterium* spp.	0.84 × 10^2^
DSS-Ω3	MLN	*Peptostreptococcus tetradius*	6.9 × 10^3^
DSS-Ω3	MLN	*Lactobacillus* spp.	0.58 × 10^2^
DSS-S	Liver	*Lactobacillus* spp.	1.82 × 10^2^
DSS-S	MLN	*Peptostreptococcus* spp.	0.54 × 10^2^
DSS-S	MLN	*Lactobacillus* spp.	2 × 10^2^
DSS-S&Ω3	Liver	*Peptostreptococcus* spp.	10^2^
DSS-S&Ω3	MLN	*Fusobacterium* spp.	2 × 10^2^

## Data Availability

Data are available to qualified researchers upon reasonable request to telonakos@gmail.com.

## Abbreviations

The following abbreviations are used in this manuscript:

DSS	Dextran Sodium Sulfate
DSS-S	DSS + Synbiotics
DSS-Ω3	DSS + Omega-3 Polyunsaturated Fatty Acids
DSS-S&Ω3	DSS + Synbiotics and Omega-3 Polyunsaturated Fatty Acids
UC	Ulcerative Colitis
IBD	Inflammatory Bowel Disease
DAI	Disease Activity Index
MPO	Myeloperoxidase
HCT	Hematocrit
HGB	Hemoglobin
MCV	Mean Corpuscular Volume
MCH	Mean Corpuscular Hemoglobin
RBC	Red Blood Cells
PLTs	Platelets
MLN	Mesenteric Lymph Nodes
CFU	Colony-Forming Units
EPA	Eicosapentaenoic Acid
DHA	Docosahexaenoic Acid
SCFA	Short-Chain Fatty Acids
ARRIVE	Animal Research: Reporting of In Vivo Experiments
